# Vanillin-Based Indolin-2-one Derivative Bearing a Pyridyl Moiety as a Promising Anti-Breast Cancer Agent via Anti-Estrogenic Activity

**DOI:** 10.1021/acsomega.2c07793

**Published:** 2023-02-08

**Authors:** Onur Bender, Ismail Celik, Rumeysa Dogan, Arzu Atalay, Mai E. Shoman, Taha F. S. Ali, Eman A. M. Beshr, Mahmoud Mohamed, Eman Alaaeldin, Ahmed M. Shawky, Eman M. Awad, Al-Shaimaa F. Ahmed, Kareem M. Younes, Mukhtar Ansari, Sirajudheen Anwar

**Affiliations:** † Biotechnology Institute, Ankara University, 06135 Ankara, Turkey; ‡ Department of Pharmaceutical Chemistry, Faculty of Pharmacy, Erciyes University, 38280 Kayseri, Turkey; § Department of Medicinal Chemistry, Faculty of Pharmacy, Minia University, 61519 Minia, Egypt; ∥ Department of Pharmacognosy, College of Clinical Pharmacy, Al Baha University, 65528 Al Baha, Saudi Arabia; ⊥ Department of Pharmaceutics, Faculty of Pharmacy, Minia University, 61519 Minia, Egypt; # Department of Clinical Pharmacy, Faculty of Pharmacy, Deraya University, 61111 Minia, Egypt; ¶ Science and Technology Unit (STU), Umm Al-Qura University, 21955 Makkah, Saudi Arabia; ∇ Central Laboratory for Micro-analysis, Minia University, 61519 Minia, Egypt; ○ Department of Pharmacology and Toxicology, Faculty of Pharmacy, Minia University, 61519 Minia, Egypt; ⧫ Department of Pharmaceutical Chemistry, College of Pharmacy, University of Hail, 81442 Hail, Saudi Arabia; †† Analytical Chemistry Department, Faculty of Pharmacy, Cairo University, El-Kasr El-Aini Street, ET-11562 Cairo, Egypt; ‡‡ Department of Clinical Pharmacy, College of Pharmacy, University of Hail, 81442 Hail, Saudi Arabia; §§ Department of Pharmacology and Toxicology, College of Pharmacy, University of Hail, 81442 Hail, Saudi Arabia

## Abstract

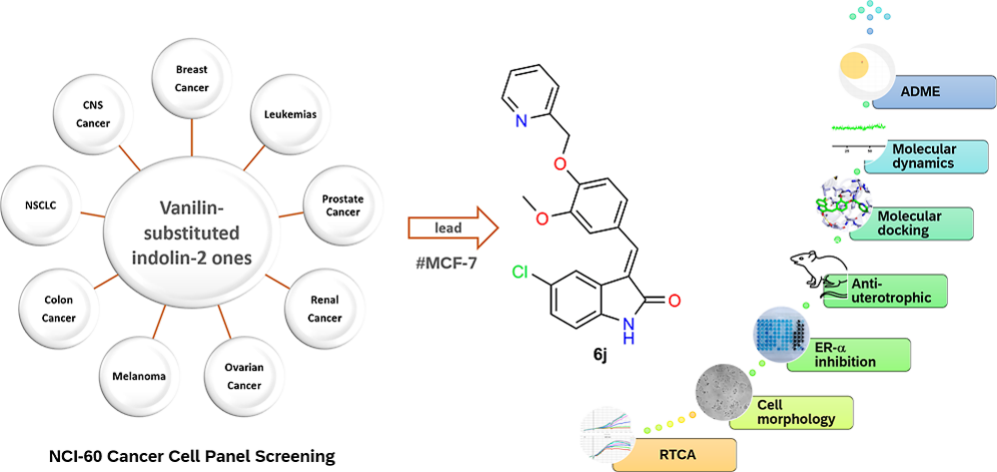

The structure-based design introduced indoles as an essential motif in designing new selective estrogen receptor modulators employed for treating breast cancer. Therefore, here, a series of synthesized vanillin-substituted indolin-2-ones were screened against the NCI-60 cancer cell panel followed by in vivo, in vitro, and in silico studies. Physicochemical parameters were evaluated with HPLC and SwissADME tools. The compounds demonstrated promising anti-cancer activity for the MCF-7 breast cancer cell line (GI = 6–63%). The compound with the highest activity (**6j**) was selective for the MCF-7 breast cancer cell line (IC_50_ = 17.01 μM) with no effect on the MCF-12A normal breast cell line supported by real-time cell analysis. A morphological examination of the used cell lines confirmed a cytostatic effect of compound **6j**. It inhibited both in vivo and in vitro estrogenic activity, triggering a 38% reduction in uterine weight induced by estrogen in an immature rat model and hindering 62% of ER-α receptors in in vitro settings. In silico molecular docking and molecular dynamics simulation studies supported the stability of the ER-α and compound **6j** protein–ligand complex. Herein, we report that indolin-2-one derivative **6j** is a promising lead compound for further pharmaceutical formulations as a potential anti-breast cancer drug.

## 1 Introduction

With 2.3 million cases and 685 000 deaths in 2020, breast cancer has become the most widespread cancer worldwide. Breast cancer comprises various genetic and epigenetic factors with explicit clinical implications.^1,2^ Different types of breast cancer are usually described by their dependence on the estrogen receptor, ER, progesterone receptor, PR, and/or human epithelial receptor 2, HER2, with ER positive (ER+) cases accounting for 75% of all cases.^3^ Since ER receptors are dysregulated in cancer cells, they are involved in uncontrolled cell proliferation, metastasis, and cancer invasiveness.^4^ Consequently, antagonizing ER receptors is part of the first-line therapy for ER+ breast cancer cases. Tamoxifen was an ER antagonist initially adopted as a targeted therapy to prevent the estrogen-stimulated proliferation of breast tumor cells. Nevertheless, it was promptly elucidated that tamoxifen possesses tissue-selective agonist traits.^5,6^ This partial agonistic activity restrains antagonism, puts the therapeutic effectiveness of tamoxifen into question, and might explain some of tamoxifen’s adverse effects.^7^ These negative effects were mitigated by developing second- and third-generation ER antagonists, currently called selective estrogen receptor modulators (SERMs).^8,9^ SERMs share a potent ER antagonistic profile in breast tissue, protecting bone tissue without a uterotrophic profile.^10,11^

**Figure 1:**
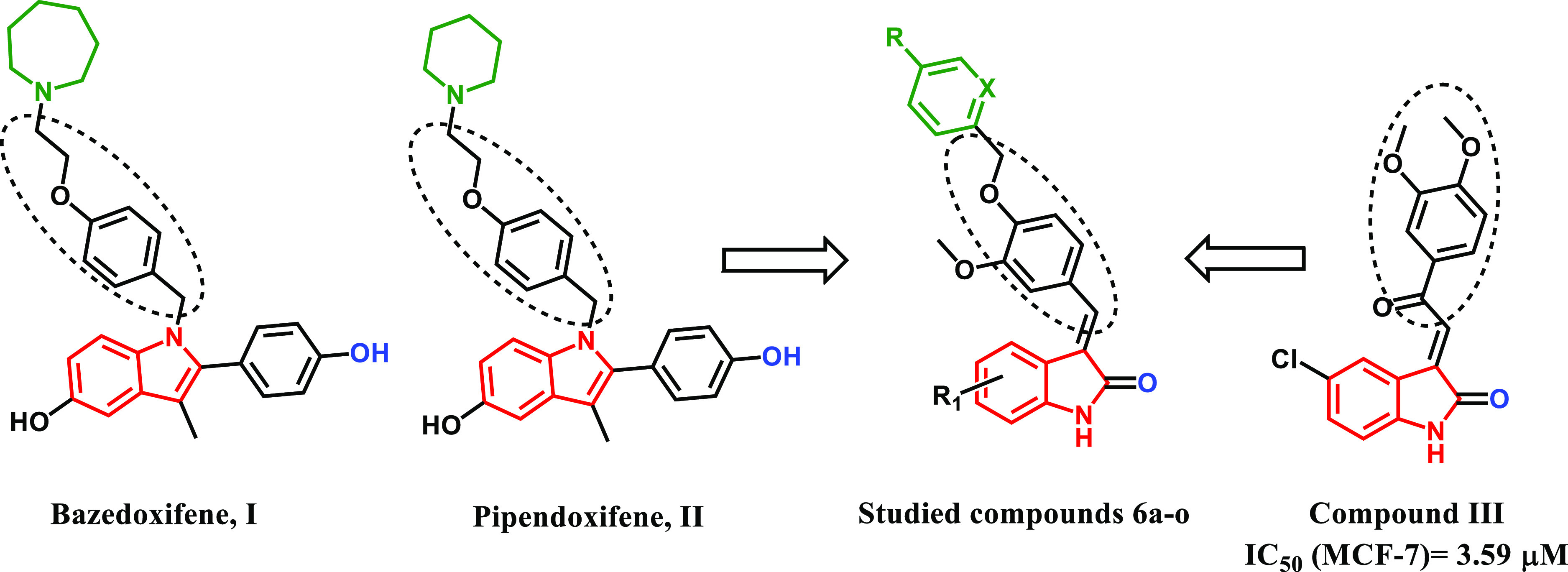
Structures of indole-based SERMs bazedoxifene I, pipendoxifene II, the previously reported anti-breast cancer indole derivative compound III, and the studied compounds **6a–o**.

Different heterocycles were introduced during the development of the second and third generations of SERMs, such as the benzothiophene-based raloxifene^12^ and the indole-based bazedoxifene.^13^ The use of nitrogen-containing heterocycles may induce a polarized behavior that contributes to establishing an efficient interaction with ER-α receptors.^14−16^ The third generation of SERM, bazedoxifene, I, (Figure 1), is an indole-based modulator approved in 2013 to treat and prevent postmenopausal osteoporosis^17^ with several current trials for application in breast cancer^18,19^ and schizophrenia.^20^ It was designed by replacing the benzothiophene core of raloxifene with an indole ring.^18,21^ It showed tumor suppressor activity in ER+ breast cancer patients.^21^ It held the potential to counteract the acquired hormonal resistance observed with other SERMs in breast cancer cell lines.^22^ It even induced anti-proliferative activity in triple-negative breast cancer via decreasing the expression of p-STAT3 and inhibiting IL-6/GP130 pathways.^23^ Such effects contribute to anti-tumor effects observed in non-hormone sensitive cancer cell lines such as head and neck^24^ and gastric and pancreatic.^23,25^ Subsequently, bazedoxifene was used as a template for designing several potential anticancer agents with the ability to modulate estrogen activity for use in breast cancer cell lines as shown in Figure 1.^26^ Furthermore, indole using is not confined to SERMs but also widely goes to the design of several anticancer agents.^27^ It can be seen in several anticancer drugs, such as sunitinib, anlotinib, osimertinib, and other agents in clinical trials such as semaxinib.^28,29^ Additionally, indole and its derivatives, such as isatin, are functional motifs in the design of anti-cancer agents with diverse mechanisms.^30^ They can evoke an anti-cancer profile by inhibiting tubulin polymerization, some tyrosine kinases (such as Akt, EGFR, and ALK), the HDAC enzyme, and topoisomerase.^30−32^ Multiple indole derivatives were also designed to target breast cancer cell lines.^33,34^ Indolin-2-one was merged with a chalcone pharmacophore to produce a series of 3-(2-oxo-2-phenylethylidene)indolin-2-ones (**6a–o**, Figure 1) that considerably inhibited the proliferation of MDA-MB-231, MDA-MB-468, and MCF-7 breast cancer cells with IC_50s_’ of 8.54, 4.76, and 3.59 μM, respectively.^35^ Merging the pharmacophore of the SERMs bazedoxifene, I and pipendoxifene, II with the previously reported anti-cancer compound III, herein, we focus the biological activity of synthesized indole-2-one derivatives **6a–o** for potential synergism of the anti-breast cancer activity observed in both compounds while retaining the inhibition of the ER-α receptor.

## 2 Results

### 2.1 Chemistry

**Scheme 1:**
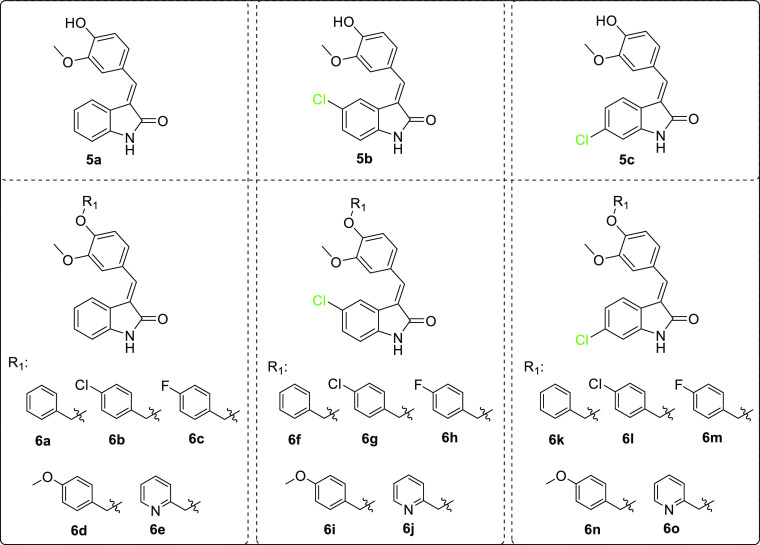
Compounds **5a–c** and **6a–o**, Which Have Been Previously Synthesized and Screened for Anti-Cancer Activity with the NCI-60 Cancer Cell Line Panel in This Study

The route for the synthesis of oxindoles **5a–c** and **6a–o** has been previously reported by our group and is summarized in Scheme 1.^36^ Vanillin or alkylated vanillin derivatives reacted with different oxindole derivatives, yielding compounds **5a–c** and **6a–o**, respectively. The resulted compounds were a mixture of E and Z isomers and used without separation as the previous literature reported that the E isomer is mainly the major isomer^37−39^ with the possibility of interconversion between the two isomers in methanol within 2 days.^40,41^ Compounds’ identities were confirmed by comparing mp and NMR data to those we had previously reported.^36^

### 2.2 NCI-60 Cell Line One-Dose In Vitro Cytotoxicity Screening

Compounds **5a–c** and **6a–o** were tested using standard NCI protocols for in vitro activity at the National Cancer Institute (NCI, Bethesda, Maryland, USA), wherein compounds were tested using one single concentration of 10 μM against 60 cell lines of nine different cancer types. The results are expressed as growth inhibition (GI, %) and listed in Table 1. Data showed a weak to moderate activity against leukemia, the central nervous system (CNS), melanoma, ovarian, renal, and prostate cancers. Excellent activity was observed against a single NSCLC cell line, EKVX, for compounds **5a–c** (GIs = 77–86%, Table 1) with no observed activity for indoles with substituted vanillin **6a–o**. Similarly, compounds **5a-o** showed good activity against the SNB-75 CNS cancer cell line (GIs = 50–59%, Table 1) with a very weak activity for compounds **6a–o**. The results also revealed excellent activity of compounds **6g** and **6h** against ovarian cancer cell lines OVCAR-3 and OVCAR-4. The GI observed was highest and ranged from 74 to 97%. All tested compounds exhibited a consistent inhibition against the MCF-7 breast cancer cell line with GIs of 6–63%. Compound **6j** showed the highest activity with a GI of 63%, while compounds **5b–c**, **6e**, **6g–h**, and **6l** showed moderate activity with GIs of 50–65%. The MCF-7 cell line was selected for further testing since it demonstrated the only consistent activity.

**Table 1:**
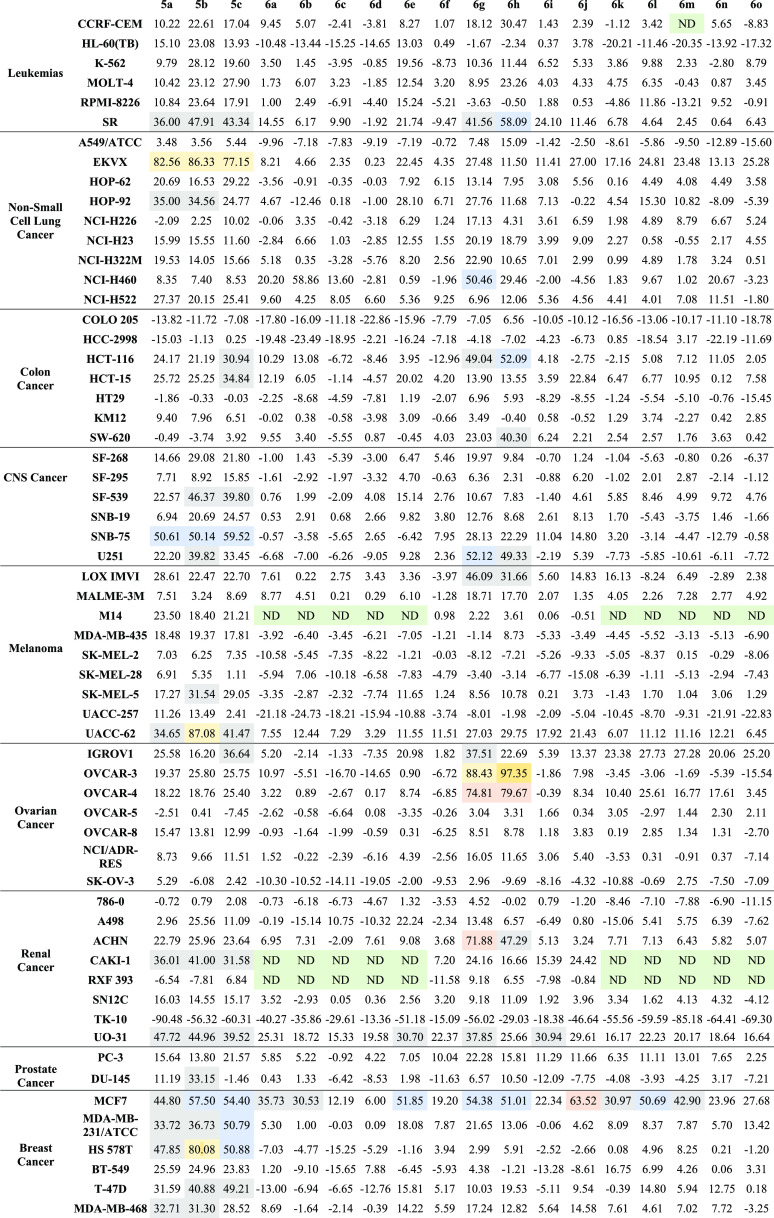
GI (%) Induced by 10 μM of Compounds **5a–c** and **6a–o** against the NCI-60 Cancer Cell Line Panel^a^

a Cells shaded green = non determined activity; cells shaded gray for GI >30%; cells shaded blue for GI >50%; and cells shaded orange for GI >60%.

### 2.3 Real-Time Cellular Analysis against the MCF-7 Breast Cancer Cell Line and MCF-12A Normal Breast Cell Line

**Figure 2:**
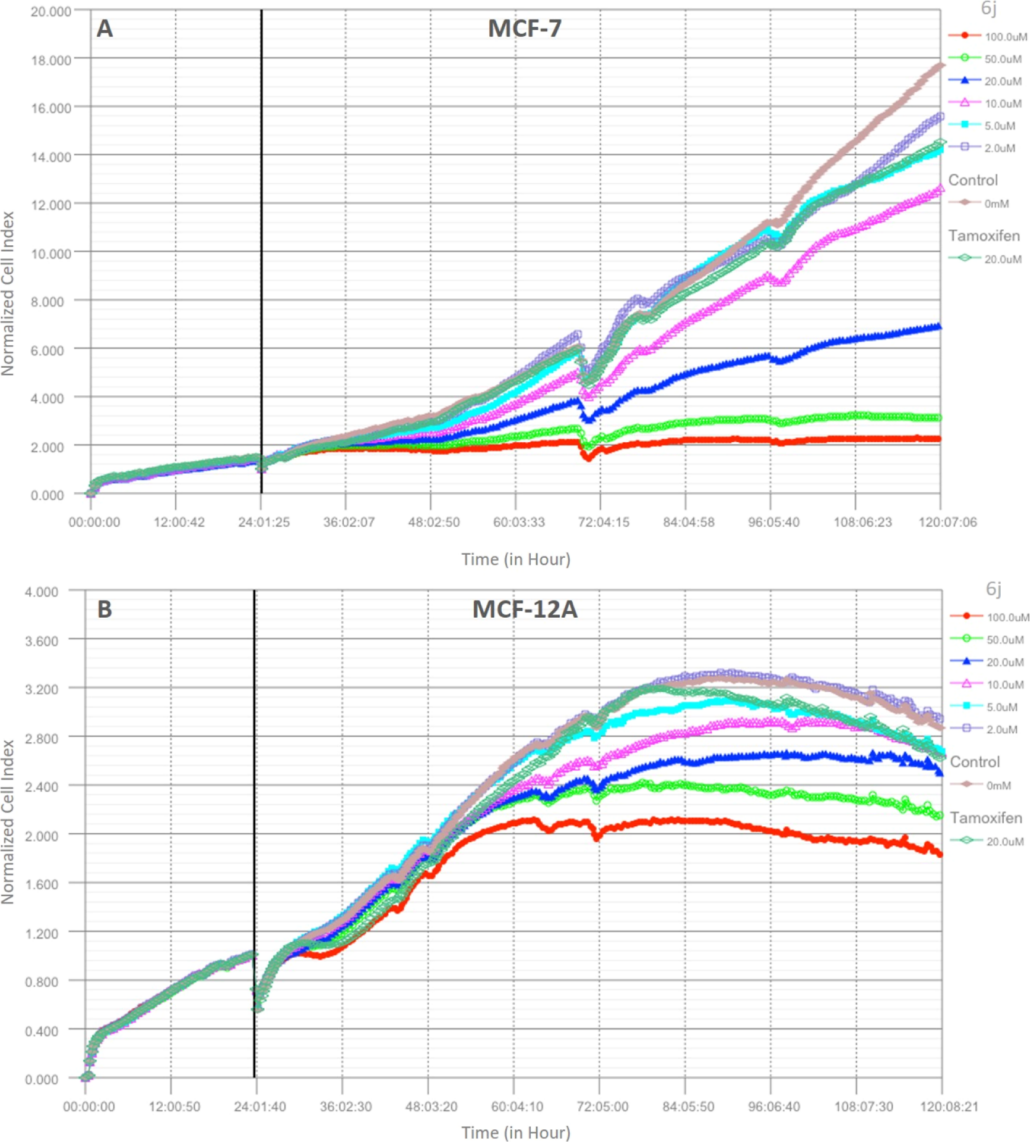
Dynamic monitoring of the effects of compound **6j** on MCF-7 and MCF-12A cells with the iCELLigence real-time cell analysis system (A) MCF-7 and (B) MCF-12A cell lines.

Since compound **6j** displayed the most potent anti-breast cancer activity against MCF-7 cells in the NCI-60 panel, we aimed to further investigate the effects of compound **6j** by using the iCELLigence real-time cell analysis system. Therefore, compound **6j** was applied in serial doses (2, 5, 10, 20, 50, and 100 μM) to MCF-7 cells and parallel to MCF-12A cells to determine the selectivity and safety. The treatments were performed 24 h after seeding the cells on system-specific biosensor-based plates. A total of 120 h of analyses were monitored, with cell viability measurements taken every 15 min. Figure 2a,b shows the results as a normalized cell index graph. IC_50_ values at 24, 48, 72, and 96 h after compound treatments were calculated by the iCELLigence software and are given in Table 2. In addition, the viability percentage values for each dose in these periods were calculated and are summarized in Table 3 for MCF-7 and Table 4 for MCF-12A. As seen in Figure 2a, compound **6j** completely inhibited the growth of MCF-7 cells at all time points at concentrations of 100 and 50 μM. In these treatments, cells were not killed dramatically after adding the highest two doses of **6j** (100 and 50 μM); instead, they entered the stationary phase. 20 μM of **6j** approximately inhibited the cell growth of MCF-7 cells at 50% in all time points, while 10 μM of **6j** inhibited the growth of MCF-7 cells by 25%. The same GI curves were observed in cells treated with 5 μM **6j** and 20 μM tamoxifen. Treatment with 2 μM of **6j** was ineffective compared to the other doses, but it slightly reduced the cell proliferation compared to that of the control. IC_50_ values after the **6j** treatments were calculated as 120.86 μM at 24 h, 16.19 μM at 48 h, 17.01 μM at 72 h, and 16.12 μM at 96 h.

**Table 2:** IC_50_ and *R*^2^ Values Obtained from Different Time Points Following Compound **6j** Treatments to MCF-7 and MCF-12A Cell Lines

	MCF-7		MCF-12A	
time points (h)	IC_50_ value (μM)	*R*^2^	IC_50_ value (μM)	*R*^2^
24	120.86	0.9962	506.11	0.9298
48	16.19	0.9998	81.87	0.9828
72	17.01	0.9951	311.68	0.9960
96	16.12	0.9959	206.87	0.9831

**Table 3:** MCF-7 Cell Viability (%) at Different Time Points after Treatment with Different Concentrations of **6j** or Tamoxifen (Relative to Control)

	cell viability (% ± SEM)							
		compound **6j**						tamoxifen
time point (h)	control	100 μM	50 μM	20 μM	10 μM	5 μM	2 μM	20 μM
24	98.36 ± 0.00	57.46 ± 0.02	62.66 ± 0.00	70.25 ± 0.02	80.00 ± 0.01	84.83 ± 0.01	91.38 ± 0.01	86.84 ± 0.02
48	97.46 ± 0.02	33.25 ± 0.02	43.36 ± 0.00	64.29 ± 0.02	76.59 ± 0.01	83.01 ± 0.02	91.38 ± 0.01	86.84 ± 0.02
72	96.91 ± 0.03	20.25 ± 0.01	27.99 ± 0.00	51.20 ± 0.01	76.59 ± 0.01	83.01 ± 0.02	91.38 ± 0.01	85.59 ± 0.04
96	96.91 ± 0.03	13.43 ± 0.00	18.43 ± 0.00	40.49 ± 0.00	74.16 ± 0.00	82.54 ± 0.01	90.67 ± 0.01	80.31 ± 0.07

**Table 4:** MCF-12A Cell Viability (%) at Different Time Points after Treatment with Different Concentrations of **6j** or Tamoxifen (Relative to Control)

	cell viability (% ± SEM)							
		compound **6j**						tamoxifen
time point (h)	control	100 μM	50 μM	20 μM	10 μM	5 μM	2 μM	20 μM
24	99.43 ± 0.01	82.05 ± 0.00	90.25 ± 0.01	93.58 ± 0.00	97.22 ± 0.01	99.06 ± 0.01	98.70 ± 0.00	84.00 ± 0.01
48	98.78 ± 0.00	67.30 ± 0.00	77.81 ± 0.01	81.96 ± 0.01	88.48 ± 0.02	93.73 ± 0.02	97.43 ± 0.02	84.00 ± 0.01
72	98.78 ± 0.00	62.63 ± 0.01	72.70 ± 0.01	80.57 ± 0.01	87.93 ± 0.02	92.74 ± 0.01	97.43 ± 0.02	84.00 ± 0.01
96	98.78 ± 0.00	61.26 ± 0.00	72.54 ± 0.01	80.57 ± 0.01	87.93 ± 0.02	92.17 ± 0.00	97.43 ± 0.02	84.00 ± 0.01

In contrast to MCF-7 breast cancer cells, **6j** displayed no effects on MCF-12A healthy breast cells during the first 24 h of treatment at any dose. It also exhibited a 5 times’ safer profile than that of MCF-7 at the 48th h. After 48 h, based on the IC_50_ values (506.11 μM at 24 h, 81.87 μM at 48 h, 311.68 μM at 72 h, and 206.87 μM at 96 h), the cells started to recover, and the safer profile continued afterward.

### 2.4 Morphological Assessment of **6j**-Treated MCF-7 and MCF-12A Cell Lines

**Figure 3:**
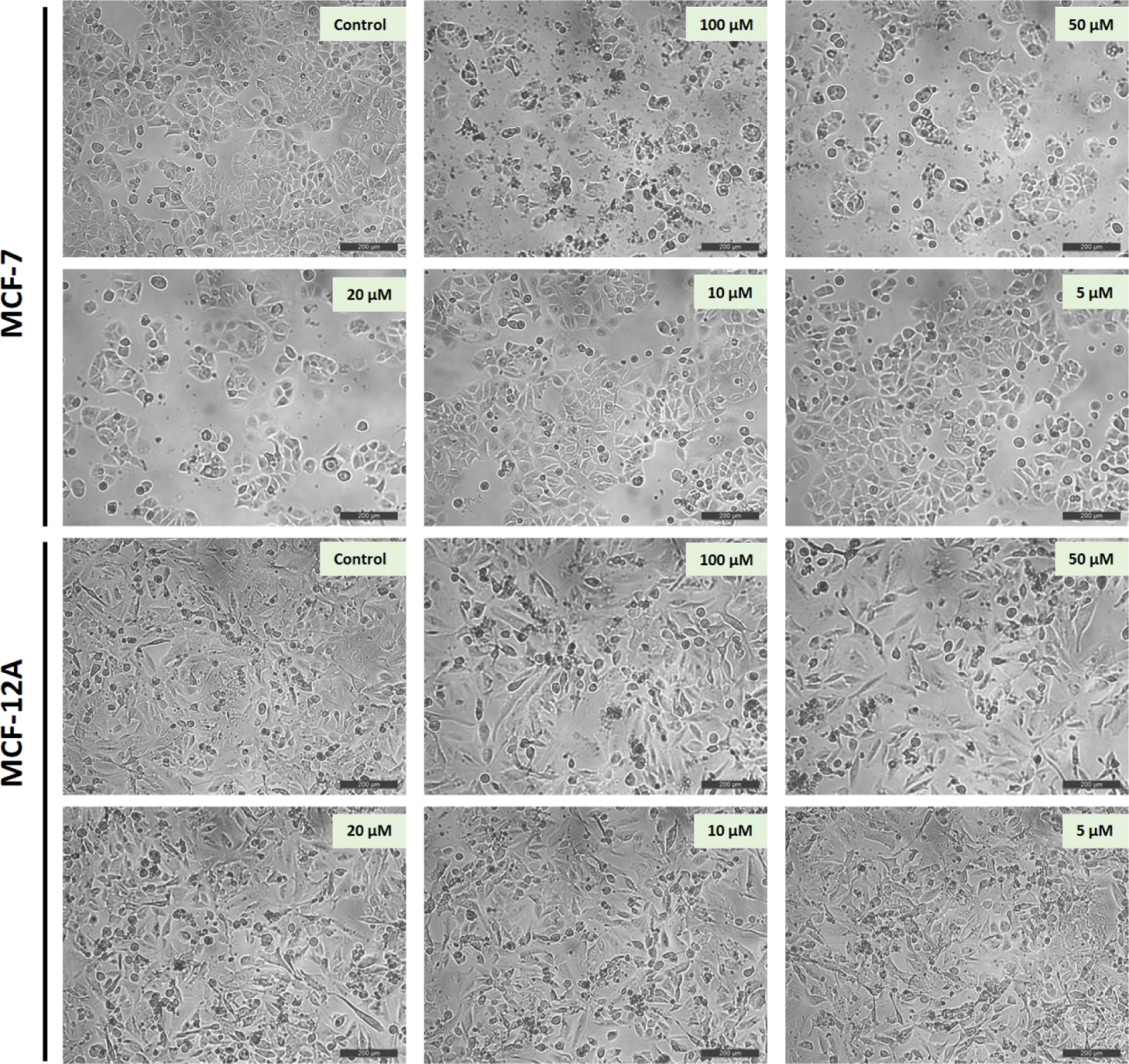
Effects of different concentrations of **6j** on the MCF-7 and MCF-12A cell morphology photographed under an inverted microscope 48 h after **6j** treatment. Scale bar represents 200 μm.

In addition to the viability analyses, morphological evaluations were performed after treating the cells with different doses of **6j** (5, 10, 20, 50, and 100 μM) to better understand what was going on in the plate wells. The cells were photographed under an inverted microscope 48 h after **6j** treatment. Figure 3 shows the effects of a 48 h treatment of **6j** on MCF-7 and MCF-12A cells. Consistent with the iCELLigence GI curves, at 50 and 100 μM doses, MCF-7 cells remained stable by stopping cell division without being toxic, but MCF-12A cells continued to proliferate. 20 μM of **6j** primarily inhibited the growth of the MCF-7 cells, while the cells displayed a healthy phenotype. The cell morphology has not deteriorated, and the membrane structures were preserved in a healthy way in the **6j**-applied cells. Compared to the control group of MCF-7 cells, no decrease in cell number was observed in MCF-12A cells treated with **6j**, especially at doses of 20, 50, and 100 μM.

### 2.5 In Vivo Anti-Estrogenic (Anti-Uterotrophic) Activity of Compound **6j**

**Figure 4:**
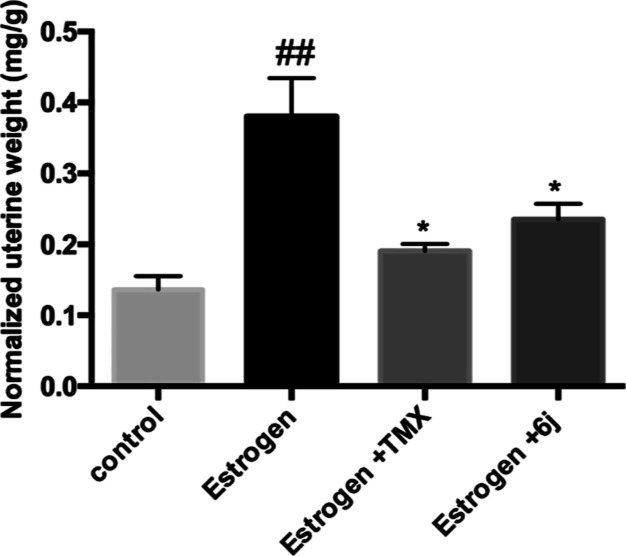
Bar chart showing the in vivo antiestrogenic activity of tamoxifen (TMX) and compound **6j**. ## denotes a significant difference from the control group at *p* < 0.01 * denotes a significant difference from the estrogen group at *p* < 0.05.

As shown in Figure 4, the anti-uterotrophic activities of tamoxifen and **6j** are expressed as normalized uterine weight and were calculated upon orally treating the rats with each compound (20 mg/kg) over three independent experiments. Estrogen alone caused a significant increase in uterus weight compared to the control, while both tamoxifen and **6j** significantly inhibited the estrogen-induced uterotrophic effect. The measured anti-uterotrophic activity of **6j** was 38% compared to that of tamoxifen (50%).

### 2.6 In Vitro ER-α Inhibitory Activity of Compound **6j**

The in vitro inhibitory activity of compound **6j** against ER-α was measured via ELISA assay to confirm the observed anti-estrogenic activities of compound **6j**. Compound **6j** inhibited 62% of ER-α activity on MCF-7 cells compared to 71% for tamoxifen. The results are listed in Table 5.

**Table 5:** Concentration of the ER-α Receptor in MCF-7 Cells Treated with Compound **6j** or Tamoxifen Compared to the Control

	results	
compound	ER-α pg/mL (mean ± SEM)	inhibition (%)
**6j**	424.9 ± 17.2	62.4
tamoxifen	327 ± 16.7	71.1
control	1131 ± 49.7	0

### 2.7 Evaluation of Physicochemical Parameters

**Figure 5:**
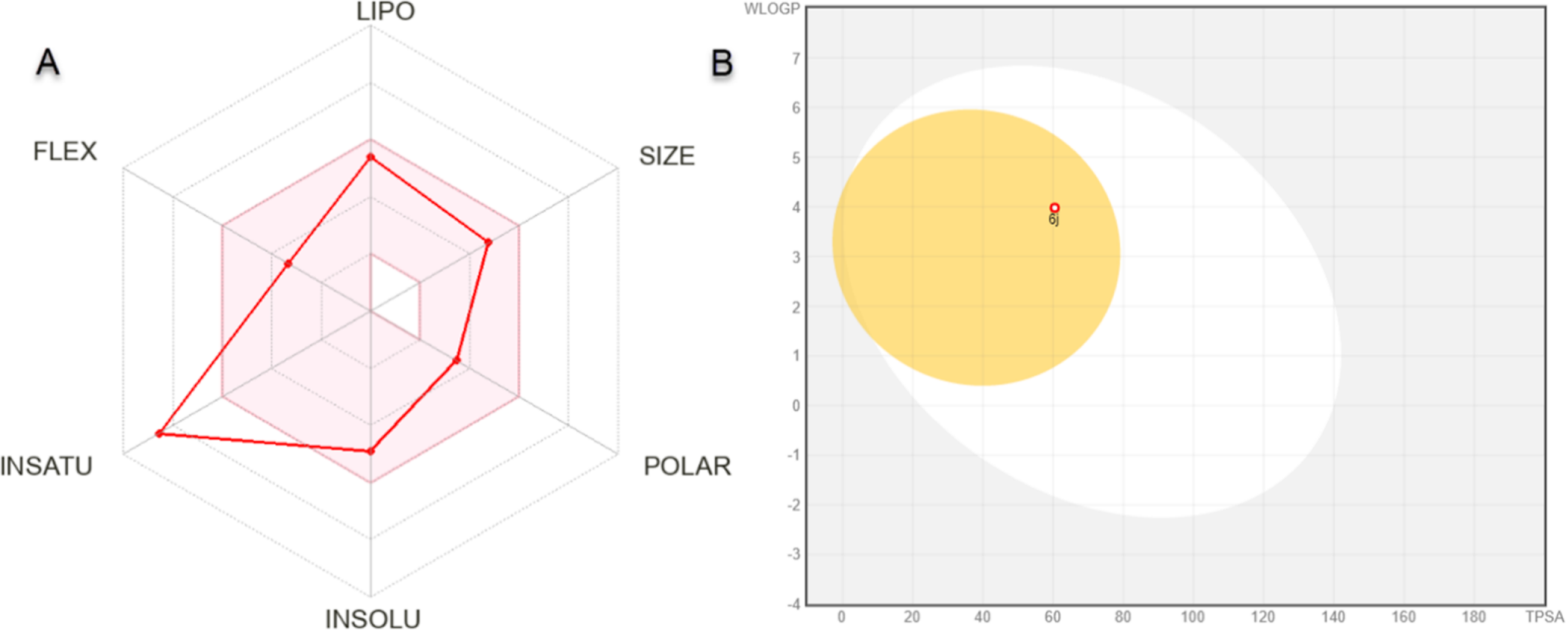
(A) Radar plot and (B) BOILED-Egg diagram obtained from the SwissADME server of compound **6j**.

Studying drug solubility is a crucial part of the pre-formulation study. It is an integral phase that every drug has been through in any development process to determine its bioavailability and the best excipients used during formulation. The solubility of compound **6j** was detected using HPLC in methanol, ethanol, and acetonitrile. Unfortunately, the method used did not detect any water solubility for **6j**, while its solubility in organic solvents ranged from 55 to 58 mg/mL. In detail, HPLC detected the solubility for **6j** to be 55.04 mg/mL in methanol, 57.33 mg/mL in ethanol, and 58.73 mg/mL in acetonitrile. With such results, compound **6j** requires the addition of a surfactant to increase its solubility, especially in water. Different types of surfactants could be used to study their effect on increasing solubility in future plans. We see that this compound has the potential to go through the formulation study. Additionally, it is a fact that some drug molecule candidates are not approved as drugs, although they are active due to their poor absorption, distribution, metabolism, and excretion (ADME) properties. Estimating these properties of synthesized compounds as in silico is a useful approach in terms of medicinal chemistry.^42,43^ Accordingly, the active compound **6j** was analyzed with SwissADME. The physicochemical properties of compound **6j**, molecular weight (392.83 g/mol), fraction Csp3 (0.09), rotatable bonds (5), H-bond acceptors (4), H-bond donors (1), molar refractivity (112.41), and topological polar surface area (TPSA) (60.45 Å^2^) were measured. For lipophilicity, log *P*_o/w_ (XLOGP3) (3.93), log *P*_o/w_ (WLOGP) (3.98), log *P*_o/w_ (MLOGP) (2.66), log *P*_o/w_ (SILICOS-IT) (5.03) and consensus It was measured as log *P*_o/w_ (3.75). For water solubility, log *S* (ESOL) is in the moderately soluble class with a value of −4.90. The ADME radar plot of **6j** is shown in Figure 5A. The colored area in this plot indicates that the compounds are in the appropriate range for predicted oral bioavailability. In terms of pharmacokinetics, compound **6j** has high gastrointestinal absorption, it is blood–brain barrier (BBB)-permeant, it is not a substrate of P-glycoprotein, and it is an inhibitor of CYP1A2, CYP2C19, CYP2C9, CYP2D6, and CYP3A4. In Figure 5B, the BOILED-Egg diagram obtained by comparing WLOGP and TPSA of **6j** is shown. This diagram shows that **6j** was passively permeable from the BBB, passively absorbed from the gastrointestinal tract, and was not effluated from the CNS by the P-glycoprotein if it was a red dot. The drug-likeness status of **6j** was detected as suitable according to Lipinski, Ghose, Veber, Egan, and Muegge’s limited rules.^44^ Considering all these parameters and data, it is predicted that compound **6j** will exhibit a favorable ADME profile.

### 2.8 Molecular Docking Analysis

**Figure 6:**
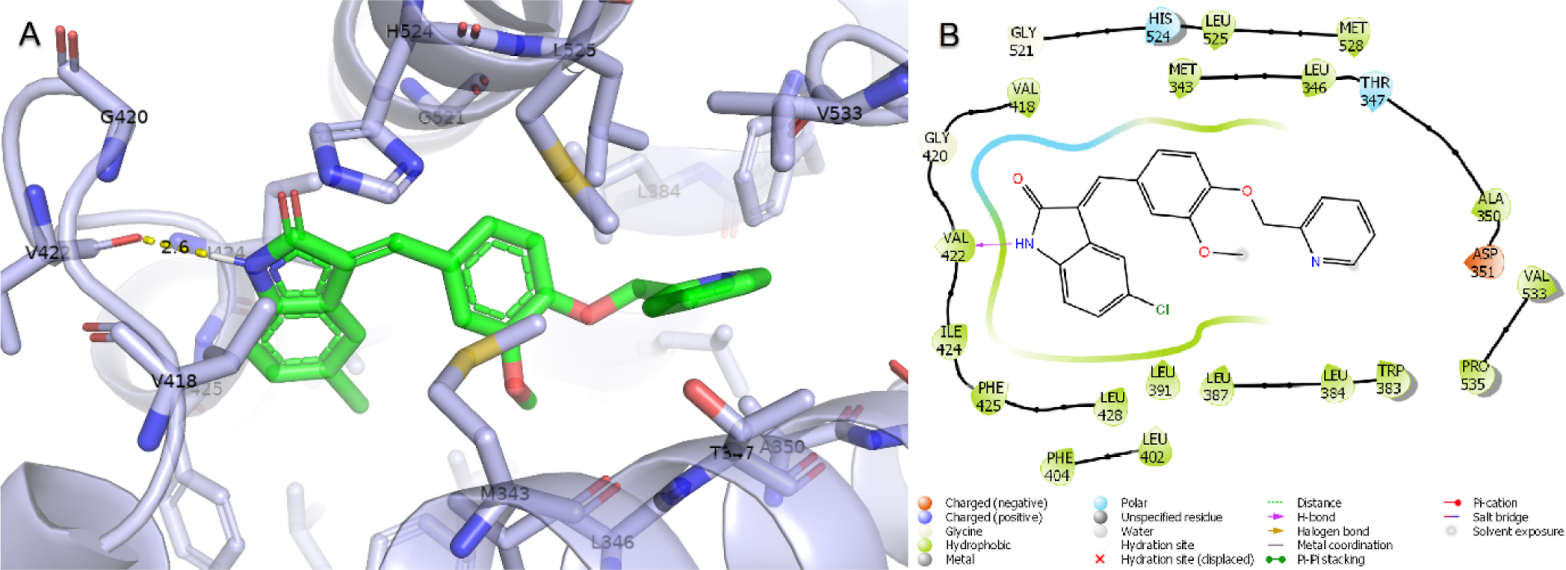
Glide molecular docking interactions of ER-α with compound **6j**. (A) Binding pose of **6j** in an ER-α active site. (B) Protein–ligand schematic interaction diagram of the ER-α and **6j** complex. (PDB ID: 5W9C).

Molecular docking studies were performed to estimate the interaction pattern and binding energy of the active molecule compound **6j** at the ER-α active site.^45,46^ For the control of molecular docking, the cocrystal ligand located in the estrogen receptor (PDB ID: 5W9C) crystal structure was self-docking and the root-mean-square deviation (rmsd) between its natural pose and docking pose was measured as 0.58 Å.^47^ The two compounds were almost completely superimposed by the rmsd value. After docking validation, compound **6j** and standard compound tamoxifen were docked to the estrogen receptor active site. The Glide gscore value, which is the binding energy of compound **6j**, was measured as −8.225 kcal/mol and tamoxifen’s as −9.694 kcal/mol. The binding poses and protein–ligand interactions of compound **6j** were analyzed and are shown in Figure 6. Accordingly, compound **6j** has a 2.6 Å long H bond with Val422, a polar interaction with Thr347 and His524, a negative charge with Phe425, and created hydrophobic interactions with Asp351, Met343, Leu346, Ala350, Trp383, Leu384, Leu387, Leu402, Phe404, Val418, Gly420, Val422, Ile424, Leu428, Gly521, Leu525, and Met528. Tamoxifen, on the other hand, formed both a face bridge and an H bond with Asp351, a polar interaction with Thr347, His524, and Asn532, a negative charge with Glu353 and Asp351, a positive charge with Arg394, and gave hydrophobic interactions with Met343, Ley346, Leu349, Ala350, Leu354, Trp383, Leu384, Leu387, Met388, Leu391, Phe404, Val418, Ile424, Gly521, Leu525, Val553, Val534, Pro535, and Leu539.

### 2.9 Molecular Dynamics Simulations

**Figure 7:**
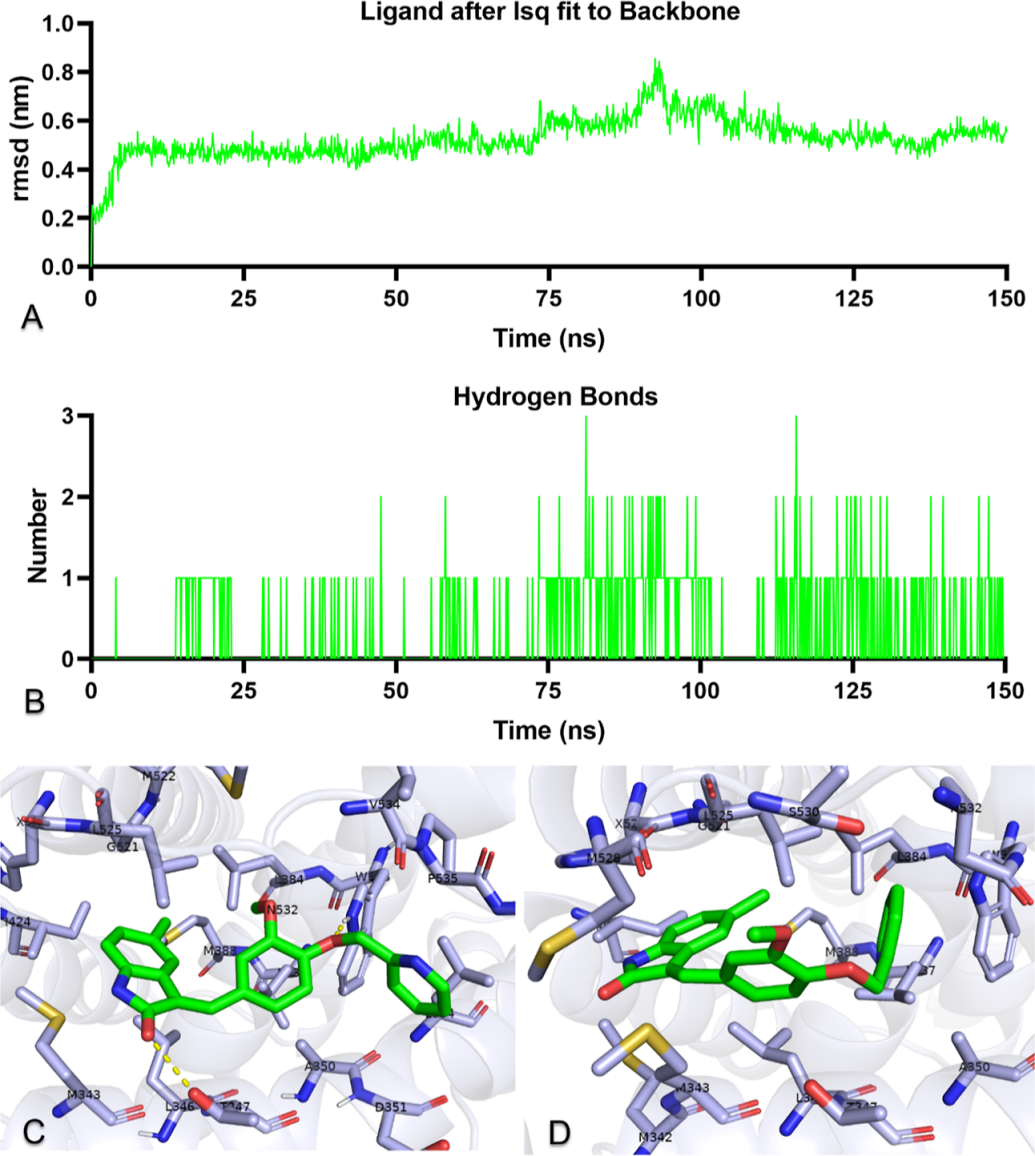
Molecular dynamics simulation trajectory analysis. (A) rmsd plot showing the stability of compound **6j** with respect to the ER-α. (B) Number of H bonds formed between compound **6j** and ER-α active site residues over 150 ns. (C,D) Binding poses of compound **6j** with ER-α at 100 and 150 ns, respectively.

To investigate and prove in silico the stability of ER-α with compound **6j**, 150 ns molecular dynamics simulations of the protein–ligand complex of ER-α and **6j** were performed.^48,49^ It is a metric that numerically shows the difference between superimposed rmsd’s and is elegantly utilized in molecular dynamics simulations. Data on the rmsd measurement obtained by fitting compound **6j** to the ER-α are shown in Figure 7A. Compound **6j** after the first 10 ns of pre-simulation is below 0.6 nm and stable up to 75 ns, with a peak up to 0.8 nm around 95 ns, below 0.6 nm after 115 ns, and stable left. The other trajectory analysis is the H-bond analysis, which expresses the change with time, showing the number of H bonds between ER-α and compound **6j**. As shown in Figure 7B, there was very sparse H bond formation in the first 15 ns, and after 15 ns, there was often one and sometimes two H bond formations.

Binding poses at 100 and 150 ns were analyzed to analyze protein–ligand dynamic interactions and changes. In Figure 7C,D, the binding modes of **6j** at 100 and 150 ns at the ER-α active site are shown. Accordingly, **6j** and Trp383 yielded one H bond (1.98 Å), Asp351 yielded a negative charge, Thr347, Ser536, and Asn532 yielded a polar interaction, and Met 343, Leu346, Ala350, Leu354, Leu384, Met388, Leu387, Ile424, Gly521, Met522, His524, Leu525, Val534, Pro535, and Leu539 yielded hydrophobic interactions at 100 ns. Compound **6j** had polar interactions with Thr347, Ser536, and Asn532 and hydrophobic interactions with Met342, Met343, Leu346, Ala350, Trp383, Leu384, Leu387, Met388, Ile424, Gkly521, His524, Leu525, and Met528 at 150 ns. In addition, an animation video was created from the molecular dynamics trajectory to monitor the protein–ligand interactions of ER-α and **6j** at the active site for 150 ns and is presented in Video S1 of the Supporting Information. It was understood that **6j** remained stable in the active site, although some interaction types and residues changed over time.

Finally, the binding free energy molecular mechanics Poisson–Boltzmann surface area (MMPBSA) formed between the protein and ligand for 150 ns was calculated from 1500 frames with the formula Δ: complex–receptor–ligand. The total binding energy MMPBSA value between ER-α and compound **6j** was calculated as–30.47 ± 1.52 kcal/mol from the sum of van der Waals, electrostatic energy, electrostatic solvation free energy evaluated from the generalized Born equation, and the nonpolar component of the solvation energy, gas-phase energy, and solvation free energy. The standard deviation here was as low as 1.52 kcal/mol and an energy value of −30.47 kcal/mol was another factor indicating protein–ligand stability.

## 3 Discussion

The use of indole-containing compounds in the fight against breast cancer is extensively described in the literature.^26,34,50^ In addition to its tubulin polymerization inhibitory activity,^51−53^ indolin-2-one has been reported to possess anti-estrogenic activity,^14,15,54^ making it an effective tool in the design of medications against breast cancer. Although there was scattered cytotoxic activity of certain compounds such as **5a–c** against some cell lines, the consistent activity of all test compounds **5a–c** and **6a–o** against the ER+ MCF-7 cell lines was similar to that of previous reports. A deeper look into the NCI in vitro anticancer screening revealed that an insignificant very weak activity was observed against ER– cells such as MDA-MB-231/ATCC with GI not exceeding 15%.

To confirm the antiproliferative activity observed against the MCF-7 cell line, the cell viability was assessed. Cell viability is regulated by biological pathways dependent on various intrinsic and extrinsic factors, and measuring the cell viability is adequately critical to the overall function and understanding of the physiology of cells. Cell viability can be measured by using several different techniques. Unlike traditional cell-based end-point assays, the xCELLigence system is a non-invasive, real-time cell analysis technology that can continuously monitor cellular dynamics, which provides more sensitive and consistent results. This technology uses electrical impedance measurement to detect cellular phenotypic changes and dynamically monitor cell proliferation via sensors.^55−57^ Also, these sensors allow the performance of a wide range of cell-based assays such as proliferation, cytotoxicity, migration, and invasion assays.^57^ Also, it distinguishes from other assays by allowing users to make the right decisions according to the current biological state of the cell before any manipulation. It eliminates the intensive steps of classical tests and risks such as being affected by some compounds due to the optical detection methods and affecting the consistency of the result.^58^

Viability results confirmed the data obtained from the NCI, and compound **6j** was able to stop the proliferation of MCF-7 cells at different concentrations and time points. Interestingly, compound **6j** showed double the activity as that observed with the use of tamoxifen at the same concentration (20 μM); **6j** induced a 30% inhibition compared to that of the same dose of tamoxifen (Table 5). These results indicate that **6j** shows cytostatic activity on MCF-7 cells without killing the cells in a toxic way as there is no significant increase in activity with increasing incubation time. Additionally, data from MCF-12A cells demonstrate the selective inhibition efficacy at all doses and periods of **6j**, Table 4 and Figure 2. In summary, **6j** shows cytostatic activity against MCF-7 ER-positive breast cancer cells, and it displays a safe profile by not showing any effect on healthy MCF-12A cells. The same conclusion was reached on examining the impact of **6j** on the MCF-7 cell morphology, Figure 3. When all these results are taken together, it has been determined that **6j** has a selective and safe cytostatic effect on MCF-7 breast cancer cells.

Investigations to further explore the mechanism of action of compound **6j** suggested its ability to block estrogen receptors. This fact was supported by NCI data mentioned earlier wherein the observed antiproliferative activity was observed only with MCF-7, which is reported to express ER+ no significant activity was identified on ER– cell lines. This assumption was supported by the ability of **6j** to antagonize the effects induced by estrogen on the rat uterus. The immature rat uterotrophic model is primarily employed to validate the impacts of estrogen agonists and antagonists on immature rats’ uteri. The model is inferred to determine the activity of a compound in the uterus quickly and accurately and can be utilized in either an agonist or antagonist mode. It depends on estrogen’s uterotrophic properties, which promote uterus development. Immature rats are employed for this test, and since they have not attained sexual maturity, endogenous estrogen has a negligible role in the estimation. After exposure to estrogen for the first time (estrone is given for 3 days), the uteri weight markedly increases as they develop quickly over these 3 days. This effect could be antagonized by co-administration of an estrogen antagonist, while estrogen agonists enhance such stimulation. Thus, the difference in uterine weight between the vehicle control and treated animals is taken as perceptive evidence of estrogen agonistic or antagonistic activities. This model successfully predicted these compounds’ clinical reactions in women.^59,60^ The results obtained by this model in the current study suggested an estrogen antagonistic activity attained by compound **6j** as it caused a 38% reduction of the uteri weight induced by estrogen, Figure 4.

The estrogen receptors primarily mediate estrogen-induced physiological process subtypes ER-α and β. An in vitro assay against ER-α supported these data with a subtype predominant in the uterus and mammary glands.^4^ ER-α is the subtype usually correlated to the development of both hormone-dependent and hormone-independent cancers. It is closely associated with cancer formation, metastasis, drug resistance, and prognosis.^61^ Thus, the ability of compound **6j** to antagonize estrogen, especially in cancer settings, was further assessed by an in vitro ER-α assay. The assay went on with the experiments mentioned above and confirmed the ability of **6j** to counteract 62% of estrogen found in MCF-7 cell lines, Table 5.

Moreover, theoretical docking studies of **6j** supported the experimental data and suggested a potential binding mode with the ER-α active site in a manner very similar to that of tamoxifen. According to in silico molecular docking and dynamic simulations, although compound **6j** and tamoxifen show close interactions, it is understood that it inhibits ER-α by showing different binding poses and interactions.

## 4 Conclusions

The current study described a series of indolin-2-one derivatives (**5a–c** and **6a–o**) as potential anti-breast cancer agents with anti-estrogenic activity. All the tested compounds exhibited weak to potent activity against the MCF-7 breast cancer cell line, where compound **6j** showed the highest observed activity with a GI of 63%. The cell viability results confirmed the data obtained from the NCI. Compound **6j** showed cytostatic activity against MCF-7 ER+ breast cancer cells. It displayed a safe profile without any significant effect on the healthy MCF-12A normal breast cell line. The results revealed that compound **6j** has a selective and safe cytostatic effect on MCF-7 breast cancer cells. Moreover, the results of the immature rat uterotrophic model and in vitro ER-α assay suggested an estrogen antagonistic activity attained by compound **6j**. Furthermore, molecular modelings are consistent with the experimental data. They predicted the potential binding patterns of the newly synthesized compound **6j** with the ER-α active site in a manner close to that of tamoxifen. Collectively, these results suggested that the herein reported indolin-2-one derivative **6j** is a promising lead compound for further optimization and development as a potentially efficient anti-breast cancer drug.

## 5 Materials and Methods

### 5.1 NCI-60 Cell Line One Dose In Vitro Cytotoxicity Screening

Anticancer activity was tested against 60 cancer cell lines at the NCI, Bethesda, USA. The screening process was done with a single dosage of 10 μM according to NCI protocols published on the NCI website https://dtp.cancer.gov/discovery_development/nci-60/methodology.htm.

### 5.2 Cell Lines and Culture Conditions

MCF-7 (Cat. no. HTB-22) (human estrogen receptor-positive breast cancer) and MCF-12A (Cat. no. CRL-10782) (human non-tumorigenic mammary epithelial) cell lines were obtained from the American Type Culture Collection (ATCC, Rockville, Maryland, USA). MCF-7 cells were maintained in Dulbecco’s modified Eagle’s medium (DMEM) (Biological Industries, Haemek, Israel) supplemented with 10% heat-inactivated fetal bovine serum (FBS) (Biowest, Nuaillé, France), 2 mM l-glutamine (Biological Industries, Haemek, Israel), 100 U/mL penicillin and 100 μg/mL streptomycin (Gibco, Waltham, MA, USA), and 2.5 μg/mL plasmocin (Invivogen, Toulouse, France) at 37 °C in a 5% CO_2_ humidified incubator. MCF-12A cells were cultured in a DMEM/F-12 Nutrient Mixture (Ham) (DMEM/F12 1:1 with HEPES and l-glutamine) (Gibco, Waltham, MA, USA) with 10% heat-inactivated FBS, 100 U/mL penicillin, 100 μg/mL streptomycin, and 10 μg/mL insulin (Humulin R, Lilly, Indianapolis, USA), 20 ng/mL epidermal growth factor (Abcam, Cambridge, UK), 0.5 mg/mL hydrocortisone (Dekort, Deva Ilac, Istanbul, Turkey), and 2.5 μg/mL plasmocin in a humidified atmosphere of 5% CO_2_ at 37 °C. The cells were routinely cultured in cell culture flasks and checked regularly under an inverted microscope. Cells reaching 80% confluency were passaged by treatment with 0.25% trypsin–EDTA. Total cell numbers were counted by the trypan blue dye exclusion method using a hemocytometer prior to the experiments.

### 5.3 Monitoring the Cellular Activities with the iCELLigence Real-Time Cell Analysis System

The iCELLigence real-time cell analysis system was used to conduct a real-time and label-free examination of the activities of compound **6j** on cells as we previously described.^62^ In brief, following a background measurement with 200 μL of complete medium on iCELLigence E-plate L8, 100 μL of MCF-7 or MCF-12A cells was seeded at a density of 5.0 × 10^3^ per well. During the 120 h monitoring, the system took impedance measurements via biosensors every 15 min. At the 24th h of incubation, the cells were treated with increasing concentrations of the **6j** compound (2, 5, 10, 20, 50, and 100 μM) in duplicate. For cell culture experiments, compound **6j** was dissolved in DMSO (Sigma, St. Louis, USA) at a stock concentration of 20 mM. For treatments, dilutions were prepared from 20 mM stock with a cell growth medium, with a final DMSO concentration of 0.1%. The medium containing 0.1% DMSO was also used as a negative control. 20 μM tamoxifen (Tocris Bioscience, Bristol, UK) was included in the study set as a positive control. Data were recorded by the iCELLigence software for 120 h and analyzed at the end of the study. The IC_50_ values at 24, 48, 72, and 96 h after the treatments were calculated using the software using six different doses’ normalized cell index values.

### 5.4 Morphological Assessment of **6j**-Treated MCF-7 and MCF-12A Cell Lines

Morphological studies were performed to observe the effects of **6j** on MCF-7 and MCF-12A cells, as previously reported.^63^ In brief, 5 × 10^5^ cells were seeded into six-well plates and incubated for 24 h. Afterward, increasing doses of compound **6j** (5, 10, 20, 50, and 100 μM) were applied to the cells. The medium containing 0.1% DMSO was used as the untreated control. 48 h after treatments, the cells were photographed under an inverted microscope, Leica DM IL LED with a DFC-290 camera (Leica, Wetzlar, Germany).

### 5.5 In Vitro ER-α Inhibitory ELISA Assay of Compound **6j**

An in vitro ER-α inhibitory ELISA assay was performed using a Human ER-α/Estrogen Receptor ELISA Kit (Sandwich ELISA) (Lifespan Biosciences, Seattle, Washington, USA) as previously described.^64^ The cells were plated at a density of 2000 cells/well in a 96-well plate. Treatment was done with 1 μg/mL of **6j** or tamoxifen in triplicate, leaving three wells as the untreated control. After 24 h, the pellets of the cells were collected by centrifugation. The cells were washed three times with PBS and then lysed by ultrasonication, and the supernatant was collected for testing. The wells were loaded with 100 μL of either standards or samples and incubated for 90 min at 37 °C. The wells were washed with 1× wash buffer for removing any unbound sample and 100 μL 1× biotinylated detection antibody was next put in and incubated for 1 h at 37 °C. The wells were rewashed with 1× wash buffer, and 100 μL 1× HRP conjugate was then added and incubated for 30 min at 37 °C. A third wash with 1× wash buffer was done and 90 μL of the TMB substrate was added. The TMB substrate reacted with the HRP enzyme, ensuring a color development, and the reaction was terminated using 50 μL of a stop solution. Finally, the optical density (OD) of the well was measured at a wavelength of 450 nm ± 2 nm. The OD of an unknown sample was calculated by correlation with a standard curve generated by standards with known concentrations.

### 5.6 In Vivo Anti-Estrogenic (Anti-Uterotrophic) Activity of Compound **6j**

The anti-estrogenic activity was assessed as previously described.^65−67^ All experiments were carried out in accordance with the recommendations of the International Animal Care and Use Committee. The experimental protocol was approved by “The Commission on the Ethics of Scientific Research”, Faculty of Pharmacy, Minia University (no. ES30/2021). 20-day-old Wistar immature female rats (40–50 g) from the animal care facility of Nahda University at Beni Suef (NUB) were allowed to acclimatize to lab conditions for 3 days before the experiment with free access to food and water. Estradiol was diluted in olive oil and subcutaneously injected on the loose dorsal skin in a dose of 10 μg/kg/day. Estradiol was diluted in olive oil and subcutaneously injected on the loose dorsal skin in a dose of 10 μg/kg/day. Tamoxifen was used in a dose of 20 mg/kg/day.^66^ Both compounds were dissolved in a mixture of DMSO, Tween 20, and saline (1:1:8, respectively) and orally administered. The rats were randomly assigned to three groups (*n* = 6) subjected to daily s.c. injections of estradiol, except for the control group. All rats receiving estradiol received an oral dose of tamoxifen or an equimolar dose of **6j** daily for 3 consecutive days, except for the control group. On the 4th day, all rats were sacrificed by cervical dislocation, and the uteri were dissected free of fat and weighed immediately. The inhibition of uterine growth compared with the growth produced by estradiol alone was used to measure the anti-uterotrophic effect. The results were expressed as percent inhibition from the formula
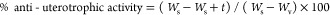
*W*_v_ = mean uterine weights from animals treated with the vehicle, *W*_s_ = mean uterine weights from animals treated with estradiol and *W*_s_ + *t* = mean uterine weights from animals treated with a combination of estradiol and the test compound. It is noteworthy that doses of the tested compounds are calculated on a molar basis. One-way ANOVA followed by Tukey’s multiple comparisons test was performed using GraphPad Prism version 6.00 for Mac (GraphPad Software, La Jolla California, USA). Values are expressed as mean ± SEM.

### 5.7 Solubility Tests and Computational ADME

The solubility of compound **6j** in various solvents (methanol, ethanol, and acetonitrile) was evaluated by adding an excess amount of the drug in a stoppered container with 0.5 mL aliquots of the used solvent. Continuous shaking was carried out in a water bath at 37 ± 1 °C for 48 h. Aliquots of the filtrate were adequately diluted with a suitable solvent and analyzed using HPLC as previously reported.^68,69^ The in silico ADME study of compound **6j** was performed via the SwissADME server (http://www.swissadme.ch/), and some physicochemical properties, lipophilicity, water solubility, pharmacokinetics, and drug-likeness properties were calculated.^44,70,71^

### 5.8 Molecular Docking

A molecular docking study was performed with the Maestro GUI of Schrödinger v2022.2.^72^ For the estrogen receptor, PDB ID: 5W9C(73) from the RCSB Protein Data Bank was selected and prepared with the Protein Preparation Wizard module by choosing OPLS4 force fields.^74^ The missing residues in the 5W9C structure were replaced with the Prime module. The 3D structure of compound **6j** and standard tamoxifen was prepared using the LigPrep module at pH = 7 ± 2 with OPLS4 force fields. Based on the cocrystal ligand in the 5W9C structure, the active site as *x*: 14.880, *y*: −11.277, *z*: −27.903, and 20*20*20 Å^3^ was created with the Receptor Grid Generation module. Molecular docking was performed with the Glide SP^75,76^ of the Ligand Docking module. 2D schematic interactions were created with the Maestro Ligand Interaction module, and the 3D binding pose was created by PyMOL Molecular Graphics System v2.4.1.

### 5.9 Molecular Dynamics Simulations

The stability of the compound **6j** protein–ligand complex with the estrogen receptor obtained by Glide SP molecular docking was tested by molecular dynamics simulation using Gromacs v2021.2.^77−79^ The files required for molecular dynamics such as solvation of the protein–ligand complex and neutralization by adding 0.15 M KCl were created with the CHARMM-GUI server (https://charmm-gui.org/).^80^ Topology files of the protein and ligand were created using Amber FF99SB.^81,82^ Molecular dynamics simulation was carried out at 300 K and 1 atm pressure. A molecular dynamics simulation with a 150 ns duration was run. The rmsd and hydrogen bond analyses of the protein and ligand were performed with gmx rmsd and gmx hbond scripts. Biding free energy MMPBSA was calculated using gmx_MMPBSA^83^ tools from 1500 frames recorded between 0 and 150 ns.

## Supporting Information Available

Molecular dynamics trajectory to monitor the protein–ligand interactions of ER-α and **6j** (MP4)
